# Chemical and sensory analysis of commercial Navel oranges in California

**DOI:** 10.1038/s41538-019-0055-7

**Published:** 2019-10-30

**Authors:** Tyler Simons, Christopher McNeil, Vi D. Pham, Siyu Wang, Yu Wang, Carolyn Slupsky, Jean-Xavier Guinard

**Affiliations:** 1 Department of Food Science & Technology, University of California, Davis, One Shields Ave, Davis, CA 95616 USA; 2 Depart of Food Science and Human Nutrition, University of Florida, Gainesville, FL 32611 USA

**Keywords:** Plant sciences, Economics, Agriculture

## Abstract

Seven lots of commercially available Navel oranges grown in California were evaluated with flavoromic, metabolomic, sensory descriptive analysis, and consumer testing techniques to identify sensory and chemical drivers of liking. Eight identified chemical clusters related to numerous sensory attributes and consumer preferences. Differences in adult and child preferences led to the discovery of six consumer clusters (four adult and two child). Sweetness, overall flavor, sourness, fruity flavor, and juiciness were identified as the main sensory drivers of liking for the consumers. Fructose, glucose, and proline were among the compounds that best explained perceived sweetness while sourness was correlated with citrate and ascorbate. Perceived fruity flavor increased with higher concentrations of ethanol. We conclude that consumers differ in their preferences for Navel oranges and desire fruit that is higher in both sweetness and sourness.

## Introduction

Fresh Navel oranges are an important part of California life. With production of the fruit valued in the tens of millions per year,^1^ the fruit plays a large role nutritionally and culturally, marked by wide consumption throughout the state. With the recent decrease in citrus production in Florida,^2^ oranges from California currently demand a premium.

Traditional breeding efforts have numerous goals, including improved fruit appearance, enhanced storage techniques, and improved texture and flavor.^3^ However, the issue of how to enhance any sensory property of the fruit is subjective and depends mainly on the parties involved in creating the novel variety. It is often that a grower or breeder may select and breed fruit that is more suited to his or her preferences. Economic viability of the new cultivar relies on consumer consumption and liking which may be disjointed from decisions that guide ease of growth. The end users should have a say as to what sensory profiles new varieties should exhibit, as they drive purchasing.

Citrus flavor is complex and difficult to characterize.^4^ The *Citrus* genus contains at least 100 unique volatile components,^5^ with dozens important to oranges.^6^ Specifically, for oranges, known important volatiles include limonene, ethyl butanoate, octanal, decanal, hexanal, (S)-linalool, and many other hydrocarbons, alcohols, aldehydes, and esters.^4,6,7^ Paired with non-volatile compounds, such as sugars and acids, these aromatic chemicals combine to evoke orange flavor. The flavor of any specific orange is also dependent on numerous confounding factors, including ripeness, waxing, and storage.^8–11^ Production practices, shipping, and other processes combine to produce fruit that arrives at market markedly different than those freshly picked from the tree.^9,12–14^ Few studies have investigated Navel oranges obtained directly from the marketplace, which best represents fruit that consumers would typically purchase.

While consumer preference studies for oranges are uncommon, past work has proved effective in ushering change for the industry. Formal research on improving oranges goes back at least to 1917 when the soluble solids concentration to titratable acidity ratio (SSC/TA) was introduced to the California citrus industry by the Department of Agriculture.^15^ Since then, work has gone on to improve chemical standards for quality, dictating a change to the BrimA measurement,^16^ a measurement based on subtracting acid from total solids content rather than using a ratio. Other work has addressed flavor from an expert standpoint,^9^ but few studies have been performed through evaluation with naive consumers.

Preference mapping is a product optimization technique that has trained panelists and consumers evaluate a set of products representative of a product category to determine preference segmentation for that category and to identify sensory drivers of liking for the uncovered preference segments.^17^ In a past study of Navel oranges, we found preference segmentation in both adults and children consumers from Northern California,^18^ where samples high in sweetness, overall flavor, orange flavor, and juiciness were preferred by both groups.

The main objective of this work was to identify chemical and sensory markers of consumer preference for Navel oranges using both chemical (flavoromic and metabolomic methods) and sensory analyses (descriptive analysis and consumer testing). Additionally, we explored a combination of penalty drop analysis via Just-About-Right scaling^19^ and consumer descriptive classification using Check-All-That-Apply^20^ for the purpose of explaining how consumers choose and appreciate Navel oranges

## Results

### Chemical analyses

Concentrations of volatile and non-volatile compounds in the Navel oranges are shown in Table 1. Nearly all non-volatile compounds were found to differ significantly among the oranges. Citrate levels were lower in Navel B than in the others. Fructose and glucose values were highest in samples A, OL, S, and F. Sucrose concentrations were highest in samples B and F. Only four volatile compounds were found to be significantly different among the orange samples: hexanal, ethylhexanoate, octanal, and linalool. Octanal and linalool showed a similar trend, with highest values in Navels OL and SW. For ethylhexanoate, Navels S and A had the highest values. Navel OL and Navel W had the highest and lowest concentrations of measured hexanal. Many of the compounds could not be compared using ANOVA as at least one sample replicate provided results that were below the detection threshold.

**Table 1:** Chemical compound concentrations (µM), detection method, and chemical cluster of the seven tested Navel oranges

Compound	Navel A	Navel B	Navel OL	Navel S	Navel F	Navel SW	Navel W	Method of analysis	Chemical cluster
Hexanal	58.88bc	89.23ab	145.65a	106.64ab	7s2.35b	78.92b	11.02c	GC-MS-GCO	1
Octanal	22.51bc	20.44bc	36.09a	11.62c	26.44ab	30.43ab	17.92bc	GC-MS-GCO	1
Linalool	251.78cd	315.92bcd	768.87a	178.39d	427.51bc	536.94ab	362.77bcd	GC-MS-GCO	1
EthylHexanoate	77.21ab	62.87ab	16.48b	125.75a	64.02ab	19.25b	23.62b	GC-MS-GCO	2
Formate	23.21a	23.96a	15.52b	24.57a	22.58a	16.47b	23.64a	NMR	2
2-Oxoglutarate	46.53bc	81.33a	37.26c	67.20ab	53.74bc	63.13ab	53.29bc	NMR	3
Adenosine	8.82bc	19.13a	8.64bc	9.84bc	8.07c	8.45bc	11.54b	NMR	3
Choline	126.32bc	217.97a	119.17c	129.89bc	144.48b	144.08b	116.49c	NMR	3
Cytidine	3.97bc	5.44a	3.38c	3.20c	3.43c	4.79 ab	3.29c	NMR	3
Limonin glucoside	1381.87bc	2227.39a	1585.94bc	1403.92bc	1621.32bc	1766.49b	1320.16c	NMR	3
Lysine	165.95b	217.89a	162.89b	158.52b	163.70b	153.76b	152.37b	NMR	3
Methanol	907.41bc	1614.76a	744.50c	1078.00b	1072.51b	778.06c	979.15bc	NMR	3
Phenylalanine	50.05bc	78.94a	41.28c	46.42bc	53.77bc	61.74b	58.61b	NMR	3
Succinate	77.61b	143.15a	66.51b	81.90b	74.37b	67.71b	86.82b	NMR	3
Tyrosine	38.76b	49.31a	25.77c	34.84b	38.26b	33.01b	36.81b	NMR	3
Uridine	11.57b	19.84a	10.84b	10.20b	9.46b	11.86b	13.00b	NMR	3
4-Aminobutyrate	1921.82b	2607.34a	1360.30c	1819.26b	2067.30b	1390.66c	1433.09c	NMR	4
Isoleucine	39.54b	49.12a	28.04d	31.99cd	37.13bc	28.09d	25.68d	NMR	4
Leucine	27.47b	38.55a	18.47c	21.38c	27.97b	19.78c	18.96c	NMR	4
Threonine	112.15ab	128.43a	96.56bcd	92.96cd	109.65bc	98.35bcd	83.09d	NMR	4
Valine	110.58ab	127.87a	72.39de	82.20cd	99.07bc	80.55d	62.88e	NMR	4
Alanine	1054.58a	673.02bc	775.11b	635.13bc	735.27bc	571.41cd	450.69d	NMR	5
Aspartate	1591.41a	1004.83bcd	1193.29abc	1342.59ab	1230.74abc	920.01cd	675.49d	NMR	5
Fructose	117416.45abc	107638.14c	118237.10abc	119376.07ab	121191.95a	108871.43bc	94281.56d	NMR	5
Glucose	121172.58a	108645.31b	122727.02a	122672.27a	124037.83a	107466.17b	93960.95c	NMR	5
Proline	10673.62b	9664.70b	9534.03b	8931.04b	13114.29a	6003.08c	5438.41c	NMR	5
proline betaine	4165.92bc	4718.31ab	4202.99bc	4112.99cd	4794.85a	3571.47de	3129.24e	NMR	5
Arginine	2721.54c	3566.17a	3046.39bc	2970.70c	3528.66ab	2615.59c	2822.93c	NMR	6
Betaine	96.43b	136.27a	111.72b	110.15b	142.63a	108.08b	115.23b	NMR	6
Ethanol	14194.86b	19864.36a	6637.88c	19846.67a	23561.01a	11282.28bc	9582.66bc	NMR	6
Sucrose	121438.03b	137246.95a	118951.31b	123754.18b	142389.49a	123647.11b	121644.95b	NMR	6
Myo-inositol	6647.85c	8153.62a	6762.25c	7627.95ab	8104.21a	7172.08bc	6743.56c	NMR	6
Ascorbate	2584.37a	2139.39b	2553.12a	2711.75a	2133.19b	2672.77a	2359.81ab	NMR	7
Citrate	42284.42a	27997.98b	39036.60a	42948.60a	38576.64a	36809.66a	41238.05a	NMR	7
Galactose	84.80c	136.19b	144.30ab	134.65b	113.79bc	177.41a	123.60b	NMR	8
Malate	5868.12cd	6732.09abc	4834.02d	4842.37d	6103.33bcd	7838.15a	7368.36ab	NMR	8
Trigonelline	31.18b	43.02a	35.38ab	36.21ab	39.71ab	42.10a	31.01b	NMR	8

The compounds were scaled and mean centered before being clustered using Euclidean distances and Ward’s Method for agglomerative hierarchical clustering—GC-MS measurements were performed on three biological fruit replicates while NMR measurements were performed on 10 biological fruit replicates. Samples sharing a letter across a row are not significant (*P* > 0.05) for that attribute by Fisher’s least significant difference test

**Fig. 1:**
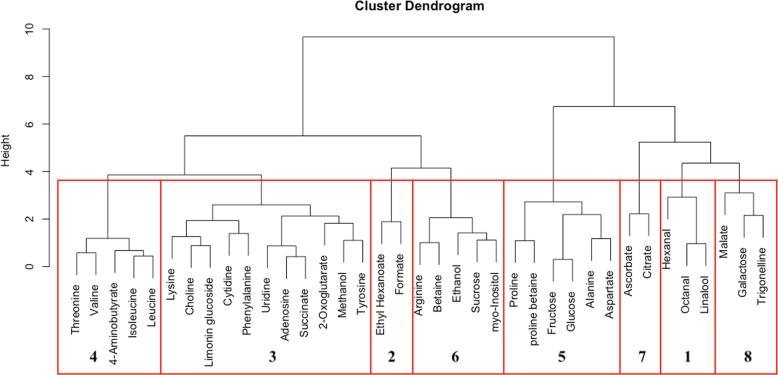
Cluster dendrogram of chemical compounds detected in the seven Navel oranges. Concentrations were scaled and centered (to mean = 0 and standard deviation = 1). A distance matrix was created between the compounds which was used to perform hierarchical clustering using Ward’s method

A distance matrix was created using the scaled (to standard deviation of 1) and mean-centered concentrations for hierarchical clustering using Ward’s Method. The dendrogram is shown in Fig. 1 indicating the presence of eight chemical clusters. The cluster groups for the compounds are shown in Table 1. Cluster 1 consisted of a few aromatic compounds, such as octanal (fruity-like) and linalool (floral-like). Cluster 5 contained fructose and glucose along with other amino acids. Ethanol and sucrose grouped into cluster 6. Citrate and ascorbate solely composed cluster 7.

### Descriptive analysis

Mean intensity ratings of the significant descriptive variables across oranges are presented in Table 2. The most intensely flavored oranges were Navels A, F, and OL while Navels B and W were the least flavorful. The highest sweetness values were given to Navels F, A, and B. The least sour samples were Navels B, W, and F.

**Table 2:** Descriptive analysis mean ratings of significantly different attributes for the seven Navel orange samples

Descriptive attributes	Navel A	Navel B	Navel F	Navel OL	Navel S	Navel SW	Navel W
Size	6.16b	5.17de	5.26d	4.74e	5.87bc	5.58 cd	7.56a
Exterior hue	6.23ab	5.61c	6.70a	6.09bc	5.62c	4.64d	6.55ab
Fruit firmness	6.04a	3.71c	4.97b	5.14b	5.02b	5.58ab	5.14b
Bumpiness	4.99a	2.11c	3.06b	4.55a	3.52b	4.55a	3.68b
Peelability	4.91c	5.00bc	6.12ab	5.04bc	5.85abc	3.13d	6.38a
Peel elasticity	4.65abc	5.46a	5.46ab	4.22c	5.55a	4.43bc	5.13abc
Segment separability	5.27a	5.61a	5.28a	5.16a	5.42a	3.79b	6.15a
Albedo quantity	5.81a	6.05a	4.66b	5.14b	5.92a	6.15a	6.14a
Interior color	5.06a	4.62abc	5.06a	4.86ab	4.45bc	4.51bc	4.15c
Interior navel size	4.01a	2.83b	1.77c	2.76b	2.93b	1.37c	3.19ab
Intensity A	6.58ab	5.39d	5.71 cd	5.92 cd	6.03bc	6.63ab	6.71a
Lemon/Lime A	2.07ab	1.51c	1.57bc	1.72bc	1.52c	2.31a	2.49a
Chemical A	2.12a	1.31c	1.16c	1.49bc	1.46c	2.07ab	2.17a
Pine A	2.05ab	1.98ab	1.65b	2.35a	1.62b	2.13ab	2.55a
Woody A	0.37b	0.31b	0.32b	0.31b	0.31b	0.29b	0.66a
Sweet	6.94b	6.74bc	7.68a	6.90b	6.31 cd	6.48bc	5.76d
Sour	2.29a	1.00c	1.67b	2.27a	2.52a	1.96ab	1.56bc
Intensity F	6.59ab	5.19 cd	6.96a	6.38ab	5.87bc	5.90bc	4.94d
Orange F	6.60a	5.24c	6.43ab	6.08ab	5.98ab	5.83bc	5.26c
Fruity F	2.28bc	3.06a	2.81ab	2.00c	1.77c	1.91c	1.74c
Juiciness	7.10a	4.74d	6.06bc	6.43ab	5.42 cd	6.19b	4.79d
Fibrousness	4.17c	5.58ab	4.26c	4.05c	5.46b	4.61c	6.25a

Thirteen judges evaluated the seven samples, blinded by three-digit codes, in triplicate, on a 10-point scale - the data was collected using FIZZ software (v2.47B, Biosystèmes, Couternon, France). Samples sharing a letter across a row are not significant (*P* > 0.05) for that attribute by Fisher’s least significant difference test

### Consumers tests

In total, 193 adults and 69 children participated in the tasting sessions. In general, adults liked the samples (Table 3), as all samples were rated above 5 = “neither like or dislike” on the 9-point hedonic scale and, except for Navel W, all were rated above 6 = “like slightly”. As a group, the most liked sample was Navel F rated around 7 = “like moderately”. On average, however, no sample exceeded 7 points. The children showed a similar trend to the adults (Table 3). No samples were significantly different except for Navel W, which was liked less than the others, not above 4 = “I like it a bit” on the 7-point hedonic scale used with the children. Both adults and children were asked how much they liked other attributes such as the flavor, texture, and appearance which were correlated to overall liking, shown in Supplementary Tables 6 and 7. These additional ratings were significantly correlated to overall liking but not all were correlated to the same degree. Liking of appearance showed the lowest correlations with overall liking while liking of flavor had the highest correlation.

**Table 3:** Overall liking values for the seven Navel orange samples by both clustered and total populations of children (*n* = 69 children, age 7–12) and adult (*n* = 193 adults, age 18+) consumer tasters—the consumers were provided with a paper ballot and randomized samples

Consumer	Cluster	Navel A	Navel B	Navel F	Navel OL	Navel S	Navel SW	Navel W
Children	1 (*n* = 26)	5.15bcd	4.27e	4.85cde	5.92a	5.77ab	5.35abc	4.62de
	2 (*n* = 43)	5.47ab	5.77a	5.98a	5.16bc	5.07bc	5.44ab	4.74c
	All (*n* = 69)	5.35a	5.2a	5.55a	5.45a	5.33a	5.41a	4.7b
Adults	1 (*n* = 62)	7.44a	6.5bc	7.31a	6.76b	5.06d	6.68b	6.16c
	2 (*n* = 84)	6.65bc	5.39d	7.15a	6.57c	7.07ab	6.68bc	4.52e
	3 (*n* = 29)	7.14a	6.79ab	5.24d	7.21a	7.1a	5.66cd	6.17bc
	4 (*n* = 18)	5.11c	7.33ab	7.72a	5.22c	6.67b	7.11ab	6.5b
	All (*n* = 193)	6.83ab	6.14d	6.97a	6.6bc	6.39cd	6.57bc	5.48e

Samples sharing a letter across a row are not significant (*P* > 0.05) by Fisher’s least significant difference test

### Just-About-Right ratings

The Just-About-Right ratings for the fruit were aggregated for all consumers on a percentage basis and are shown in Table 4 for the adults and Table 5 for the children.

**Table 4:** Just-About-Right (JAR) rating proportions for the seven Navel oranges for different taste and texture modalities as rated by the adult consumers (*n* = 193, age 18+)

Rating	Navel A	Navel B	Navel F	Navel OL	Navel S	Navel SW	Navel W
JAR sweetness							
Too little	0.25	0.46	0.33	0.41	0.48	0.43	0.57
JAR	0.68	0.45	0.61	0.51	0.46	0.50	0.36
Too much	0.06	0.08	0.07	0.07	0.07	0.07	0.07
JAR sourness							
Too little	0.33	0.58	0.37	0.40	0.34	0.43	0.57
JAR	0.58	0.34	0.54	0.51	0.55	0.48	0.34
Too much	0.09	0.08	0.09	0.09	0.11	0.09	0.09
JAR firmness							
Too little	0.27	0.31	0.23	0.30	0.17	0.23	0.33
JAR	0.68	0.65	0.73	0.66	0.78	0.73	0.62
Too much	0.04	0.04	0.04	0.04	0.05	0.05	0.04
JAR juiciness							
Too little	0.13	0.18	0.14	0.20	0.21	0.19	0.29
JAR	0.85	0.80	0.85	0.79	0.78	0.80	0.70
Too much	0.02	0.02	0.02	0.01	0.01	0.02	0.02

The adults were presented all seven navel orange samples in a randomized order and given water and crackers to cleanse their palate between samples—JAR questions were evaluated using a 5-point scale

**Table 5:** Child Just-About-Right (JAR) rating proportions for each fruit for different taste and texture modalities

Kids	Rating	Navel A	Navel B	Navel F	Navel OL	Navel S	Navel SW	Navel W
JAR sweetness	Too little	0.20	0.20	0.12	0.20	0.25	0.22	0.35
	JAR	0.68	0.59	0.75	0.65	0.62	0.70	0.48
	Too much	0.12	0.20	0.13	0.15	0.13	0.09	0.17
JAR sourness	Too little	0.19	0.49	0.26	0.28	0.25	0.36	0.46
	JAR	0.59	0.42	0.64	0.58	0.58	0.52	0.41
	Too much	0.22	0.09	0.10	0.15	0.17	0.12	0.13
JAR firmness	Too little	0.23	0.17	0.12	0.15	0.15	0.19	0.19
	JAR	0.67	0.68	0.80	0.73	0.73	0.61	0.68
	Too much	0.10	0.15	0.09	0.13	0.13	0.20	0.13
JAR juiciness	Too little	0.07	0.22	0.13	0.19	0.18	0.09	0.17
	JAR	0.70	0.65	0.74	0.68	0.71	0.71	0.57
	Too much	0.23	0.13	0.13	0.13	0.12	0.20	0.26

Children (*n* = 69 participants, ages 7–12) tasted all seven Navel orange samples and evaluated them for the JAR attributes on a 3-point scale

For the adults, the samples with the highest proportion of Just-About-Right ratings for sweetness were Navels A and F, both over 60%. Their ratings were skewed right; even the sweetest fruit showed only 8% ratings of “Too Sweet”. A similar trend was seen with JAR sourness. The samples with the highest proportion of JAR sourness were Navels A, S, and F. For all fruit, however, at least 33% of the population found them to not be sour enough. For the most sour fruit (as rated by DA), only 11% of the group found it be to be sour.

For the children, their ratings were more normally distributed, with higher proportions expressing that they found the fruit to be just right for sourness (Navels A and S). The other Navels tended to not be sour enough for them. Navel W, for example, was rated “Not sour enough” by 46% of the child population.

For JAR firmness and juiciness, the trends were similar in both adults and children. The ratings were right-skewed; the consumers thought the fruit was both not firm enough and not juicy enough. The proportions for Just-About-Right were, however, higher than for sourness and sweetness. This indicated that the texture was acceptable but all of the oranges could have been a bit more juicy and firm.

### Penalty analysis

Mean liking penalty analysis was performed using the JAR data and the overall liking values. For the adults, too little sweetness was marked frequently and had the largest impact on overall liking. Too little sourness was used slightly more often but had a lower penalty, just over 1.2 hedonic points on the 9-point hedonic scale. Too much sourness carried a large penalty as well but was used much less frequently overall.

For the children, too little sourness was used most frequently but had a relatively small penalty in comparison to too little sweetness. The children had much higher proportions of samples being rated JAR. Too little sweetness and too much sourness had the highest penalties, at just over 1.5 hedonic points (but on a 7-point hedonic scale).

### Check-All-That-Apply

The consumers also characterized the fruit using Check-All-That-Apply attributes that were generated from a mix of hedonic terms and descriptive attributes from the descriptive panel (Supplementary Tables 1 and 2). All of the CATA attributes showed significant differences between the products as shown for the adults in Supplementary Table 8.

Based on the overall liking values (Table 3) the oranges that were liked the most were Navels A and F, while the least liked samples were Navels B and W. Navels A and F had the highest values of the terms “Aromatic”, “Sweet Tasting”, “Flavorful”, “Tropical Flavor”, “Fresh”, “Complex Flavor”, “Balanced Flavor”, and “Juicy”. Navels B and W had the lowest ratings of “Sour Tasting”, “Flavorful”, “Typical Orange Flavor”, “Fresh”, “Balanced”, and “Juicy”. These samples also had the highest values of “Bland”.

### Consumer clusters and descriptive attributes

**Fig. 2:**
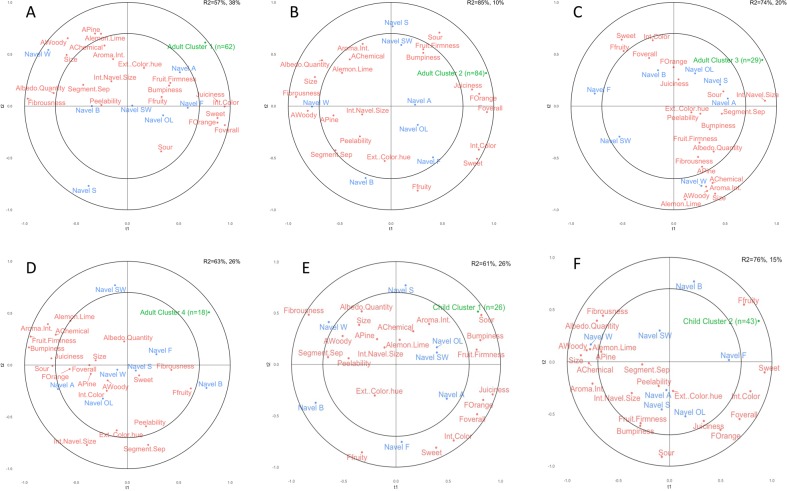
Partial least-squares 1 regression of consumer clusters onto significantly different descriptive analysis ratings. The product scores were scaled to fit to include into the regression to create a biplot. **a** Adult cluster 1 (*n* = 62) average ratings (*R*^2^ = 57%, 38% for t1, t2, respectively). **b** Adult cluster 2 (*n* = 84) average ratings (*R*^2^ = 85%, 10% for t1, t2, respectively). **c** Adult cluster 3 (*n* = 29) average ratings (*R*^2^ = 74%, 20% for t1, t2, respectively). **d** Adult cluster 4 (*n* = 18) average ratings (*R*^2^ = 63%, 26% for t1, t2, respectively). **e** Child cluster 1 (*n* = 26) average ratings (*R*^2^ = 61%, 26% for t1, t2, respectively). **f** Child cluster 2 (*n* = 43) average ratings (*R*^2^ = 76%, 15% for t1, t2, respectively)

The consumers were clustered according to their overall liking scores (Table 3). These clusters were correlated to the descriptive attributes through partial least-squares (PLS) regression (Fig. 2). The children showed two different preference clusters. The larger group of child consumers (*n* = 43) had preferences positively correlated to sweeter fruit and negatively correlated to chemical, pine, lemon/lime, and woody aromas, as in Navel samples A, B, and F. The smaller group (*n* = 26) of children preferred the sour samples, Navel OL, S, and SW.

Adult Cluster 1 (*n* = 62) was loaded positively to the first and second dimensions. These consumers preferred the Navel A and F samples, two of the samples with the highest overall flavor. The largest consumer cluster, Adult Cluster 2 (*n* = 84) was clearly driven by the overall flavor, juiciness, and orange flavor. Their preferences were negatively correlated with fibrousness and woody flavor. There were no easily identified sensory drivers of liking for the smaller clusters 3 and 4. In follow-up analyses, clusters 3 and 4 were combined but no significant relationship was found between their pooled preferences and sensory attributes as measured by the descriptive panel.

### Consumer clusters and chemical clusters

**Fig. 3:**
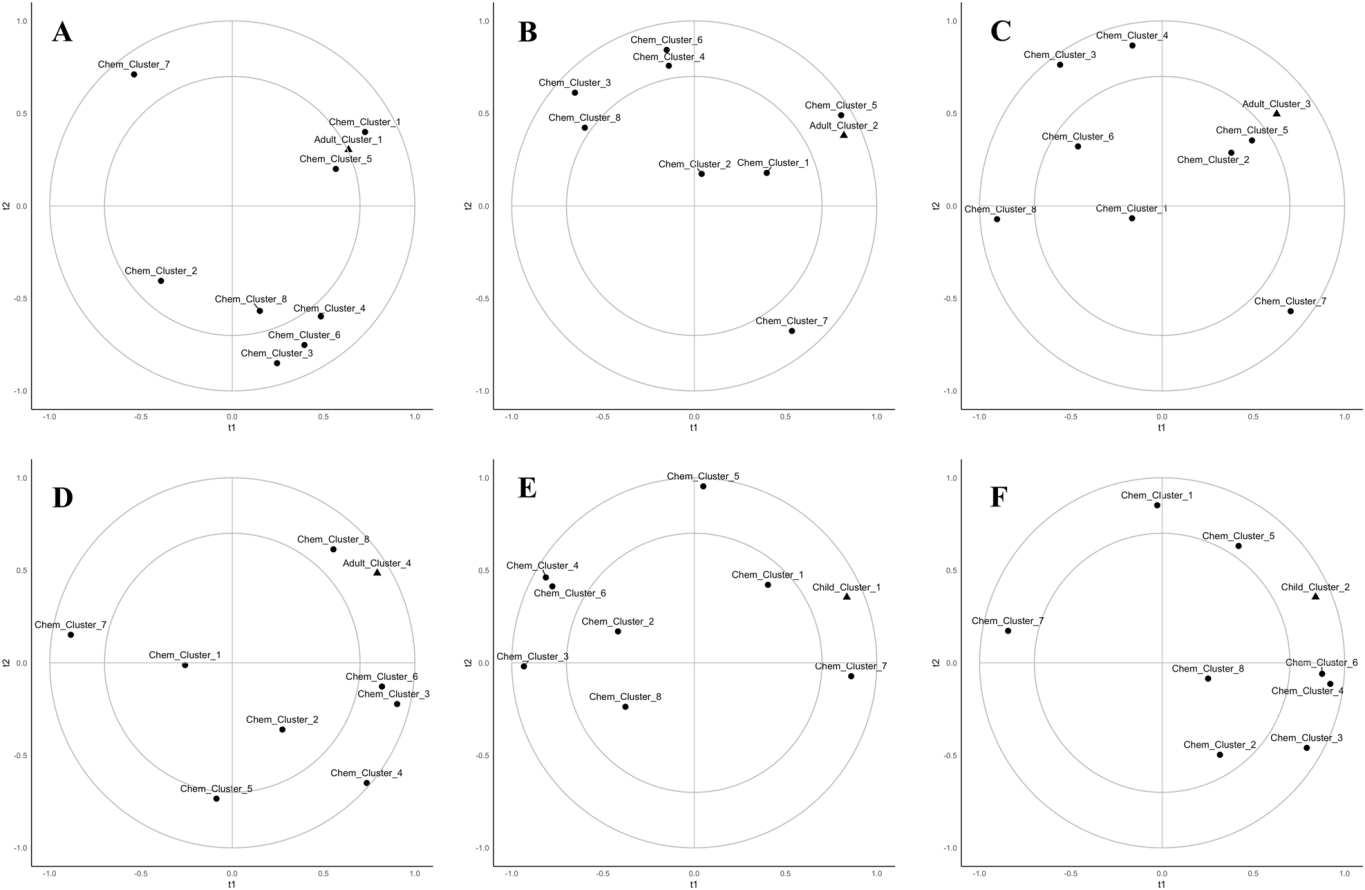
Partial least squares 1 regression of consumer clusters on scaled and centered chemical cluster averages. **a** Adult cluster 1 (*n* = 62) average ratings (*R*^2^ = 47%, 26% for t1, t2, respectively). **b** Adult cluster 2 (*n* = 84) average ratings (*R*^2^ = 76%, 4% for t1, t2, respectively). **c** Adult cluster 3 (*n* = 29) average ratings (*R*^2^ = 20%, 5% for t1, t2, respectively). **d** Adult cluster 4 (*n* = 18) average ratings (*R*^2^ = 59%, 7% for t1, t2, respectively). **e** Child cluster 1 (*n* = 26) average ratings (*R*^2^ = 84%, 7% for t1, t2, respectively). **f** Child cluster 2 (*n* = 43) average ratings (*R*^2^ = 69%, 4% for t1, t2, respectively)

One of the main goals of this work was to relate the chemical measurements performed on the groups of oranges with the consumer preference clusters that were uncovered in the analyses. Partial least-squares regression was performed on the consumer clusters with regard to the chemical cluster values. This was done for the adults (Fig. 3a–d) and for the children (Fig. 3e, f).

Within Fig. 3, the majority of consumers showed a strong correlation to higher relative values of fructose and glucose (chemical cluster 5). These compounds made up the majority of the sugar detected in the Navel oranges (Table 1). Liking was unrelated to sucrose content. In this case, aspartate, proline, and alanine were also correlated with fructose and glucose. These chemical clusters, combined with the descriptive analysis, show that the preferred sensory profiles included sweetness, overall flavor, and orange flavor, and occasionally sourness.

### Descriptive attributes and chemical clusters

**Fig. 4:**
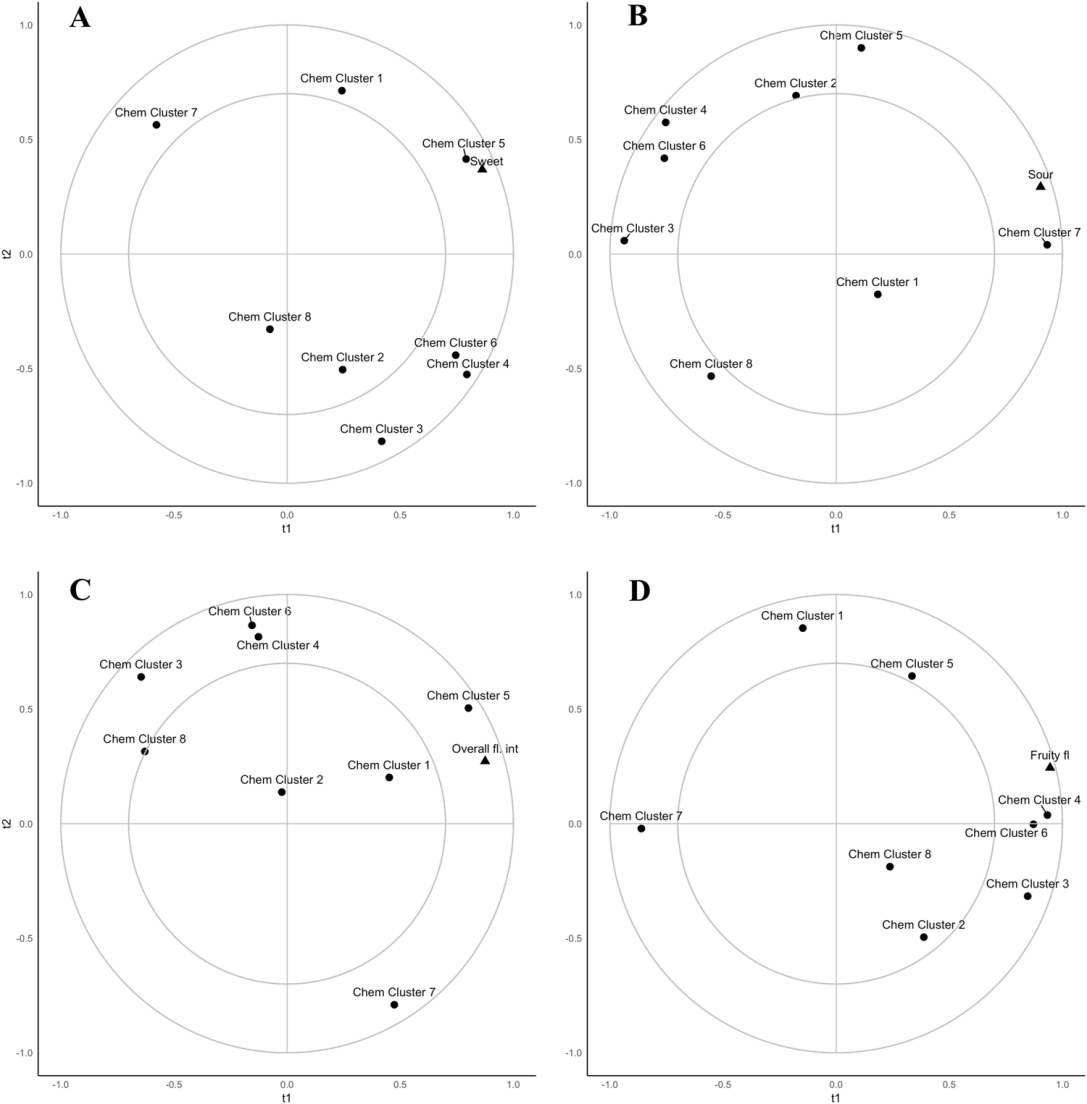
Partial least squares 1 regression of selected descriptive attributes average ratings for the seven Navel oranges on scaled and centered chemical cluster averages. **a** Sweetness (*R*^2^ = 73%, 15% for t1, t2, respectively). **b** Sourness (*R*^2^ = 80%, 15% for t1, t2, respectively). **c** Overall flavor (*R*^2^ = 83%, 4% for t1, t2, respectively). **d** Fruity flavor (*R*^2^ = 90%, 5% for t1, t2, respectively)

The chemical attributes influenced the ratings given by the descriptive panel. Sweetness was found to be strongly correlated to chemical cluster 5 (Fig. 4a), the cluster containing fructose, glucose, aspartate, proline, and proline betaine. Sourness was positively correlated with chemical cluster 7 (citrate and ascorbate, Fig. 4b), while negatively correlated to compounds in chemical cluster 8 (malate, galactose, trigonelline). Overall flavor was most strongly related to chemical cluster 5 (Fig. 4c). Fruity flavor was strongly associated with chemical clusters 4 and 6 and away from chemical cluster 7 (Fig. 4d).

## Discussion

In a previous study, it was found that Californian consumers tended to prefer Navel oranges that were high in overall flavor, sweetness, and juiciness.^18^ All four adult clusters identified in this study should be taken in context and in tandem with the last study, as consumer cluster stability is difficult to prove in sensory studies. It seems plausible that the unidentified preferences of adult clusters 3 and 4 may be due to their smaller panelist count and the overall similarity of tested Navel oranges. However, the two largest adult consumer clusters (clusters 1 and 2) preferred oranges that were rated highly in overall flavor, orange flavor, sweetness, and juiciness (Fig. 2a, b), which is in agreement in the past study.^18^ The children were more clearly split between sweetness and sourness, where the first cluster preferred the more sour fruit and the other preferred fruit that was sweet and fruity (Fig. 2e,f).These findings also support past work that has shown key attributes such as sweetness and orange flavor are often predictive of consumer liking.^21–23^

The chemical relation to descriptive attributes is clear with the attributes of fruity flavor, sourness, and sweetness. Fruity flavor was related to compounds that clustered with ethanol (Fig. 4d). Past work has found that flavor degradation can be traced to increasing concentrations of ethanol, with fruit transitioning from tart and citrusy to fruity with off flavors.^24–26^ Sweetness and sourness are usually well explained by sugars and acids in citrus.^4^ Sourness was related to citrate and ascorbate which comprised chemical cluster 7 (Fig. 4b). For sweetness, total soluble solids is often used to help predict sweetness.^16^ The findings presented here show that fructose and glucose contributed more to sweetness than sucrose (Fig. 4a), even though sucrose was found at a similar concentration to both of the monomeric sugars. In addition, the compounds that clustered with glucose and fructose, such as proline, aspartate, and alanine are amino acids that are increased in response to plant stress.^27^ Stress, such as that caused by drought,^28^ can increase synthesis of flavor compounds as well as flavonoids, which are key nutrients in oranges. The stress induced flavor compounds may have additionally influenced the perceived sweetness of the fruit through taste–odor interactions as has been shown with fruity compounds.^29^

Compounds responsible for orange flavor and overall flavor intensity were elusive. Orange flavor was not well described by the statistical models presented here. Although there was no cluster of compounds that were positively related to those attributes, chemical cluster 8, consisting of malate, trigonelline, and galactose, showed a negative relationship with orange flavor. Trigonelline has been found in higher levels in salt-stressed citrus fruits.^30^

The purpose of this study was to relate the sensory profile and consumer liking to chemical compounds. Sugars, acids, and volatiles are at the core of citrus flavor.^4,6,7,9,12,22^ The key compounds studied here affected the perceived citrus flavor, as shown by the ratings from descriptive panel. These flavor differences influenced the non-homogeneous consumer preferences clusters. While the chemical drivers of liking for the consumers varied by cluster, there were clear trends. Sweetness, driven by compounds in cluster 5 such as fructose, glucose, proline, aspartate, alanine, and proline betaine, was a major driver of liking for nearly all of the consumers. Sourness, as chemical cluster 7, was also shown as a strong driver of liking for child cluster 1. Chemicals in cluster 1, hexanal, octanal, and linalool, acted as a driver of liking for adult cluster 1. These compounds are known to be important to citrus flavor^31^ but did were not strongly related to any descriptive attribute in this study.

In order to market Navel oranges more effectively to the consumers, those words selected by the consumers in the CATA portion with a positive sentiment could be used in messaging and advertising. For example, adult cluster 1 liked Navel A (Table 3) and they also noted that this sample could be described by the terms “Good Appearance”, “Aromatic”, “Sweet Tasting”, “Flavorful”, “Typical Orange Flavor”, “Fresh”, and “Juicy” (Supplementary Table 8). Successful marketing might leverage these key words while avoiding more confusing terms such as “complex flavor” or “floral flavor”.

The consistently high quality and flavor homogeneity of oranges produced in California make it difficult to study how consumers preferences segment in accordance to different flavor profiles. However, improvement in internal fruit quality is still possible, which would boost consumer liking. Our research has shown that commercially produced Navel oranges could benefit from more sugar and acidity, based on consumer JAR (Tables 4 and 5) and CATA (Supplementary Table 8) ratings. Changes to the fruit happen during processing, waxing, shipping, and storage leading to fruit from that at the packinghouse.^14,25,32^ As the fruit presented here was not stored in true supermarket conditions, a future study of fruit throughout the entire supply chain may identify key control points in sustaining high acceptance for Navel oranges overall.

## Methods

### Fruit

Seven commercial Navel oranges from various producers in California, harvested in February 2017, were obtained from growers and packers through the California Citrus Research Board (CRB) or purchased at local grocery stores and produce wholesalers. The fruits were treated according to current industrial processing practices, including washing, rinsing, waxing, grading, and boxing.^3^ Upon reception at the University of California, Davis, the fruit was stored cold at 4 °C and 85% humidity. All fresh samples were stored for less than one month and all sensory experiments were performed within a 10-day span (3/3/17–3/10/17) to prevent sensory changes to the fruit. Samples tested were taken out of cold storage approximately 12 h before both descriptive analysis and consumer tests in order to equilibrate them to room temperature. For NMR analysis, samples were peeled, juiced using a handheld citrus press, and immediately frozen at −80 °C until processing. For gas chromatography–mass spectrometry (GC-MS) analysis, unpeeled samples were squeezed manually using a hand blender without applying heat or any solvent to obtain juice, and unused peel, pulp, and seeds were eliminated. Seven lots of fruit were collected due to the number of commercially available navel oranges on the dates of testing as well as to limit the sensory fatigue of the consumer panelists.

### Descriptive analysis

Generic descriptive analysis^17^ was performed using 13 judges (9 females, 4 males, ages 25–75), many of whom were part of a previous citrus descriptive panel.^33^ Panelists completed seven training sessions on Navel oranges. The first two sessions involved term generation based on citrus found in local retail outlets. The following sessions focused on attribute alignment, aided by the use of references, to finalize a list of descriptive terms, shown in Table 6, to ensure the judges rated the attributes in a similar manner. During training, judges used a sample ballot that listed each of the terms with an adjacent 10 cm line scale anchored at 1 cm indentations to limit scale end use effects.^17^ An electronic ballot was designed for data collection through FIZZ (v2.47B, Biosystèmes, Couternon, France) for the actual descriptive analysis. For evaluation, the judges were first presented with a group of seven Navel oranges to rate the visual attributes of the samples. For the following terms, the judges were presented with one half of one orange sample cut through the stem end, instructed to peel the half, and evaluate. This technique was performed because the panelists felt that they could not accurately score the appearance attributes of the fruit from a single piece of fruit. Unsalted crackers (Mondelez, East Hanover, NJ) and water were provided to cleanse their palates between samples. The samples were identified using random three-digit codes and evaluated in triplicate under white light. Presentation order of the samples was randomized using a William’s Latin Square design provided by the FIZZ system.

**Table 6:** Descriptive analysis attributes and references—13 judges (9 females, ages 25–75) were trained to perform generic descriptive analysis on market-available Navel oranges using a 10 cm line scale

Modality	Term	Description	Reference	anchors
Appearance	Size	Relative size of the fruit	Low, 1.5 inch diamater; high, 4 inch diameter	Low–high
	Blemished	Amount of blemishes on the peel of the fruit	Low, unblemished; high, severe scarring and blemishes	Low–high
	Exterior color hue	Hue of the peel	Low, yellow; high, dark orange	Low–high
	Uniformity of color	Relative uniformity of color of the peel over the entire fruit	Low, radical color change across the fruit; high, very uniform in color	Low–high
Texture (by hand)	Fruit firmness	Hardness of the whole fruit	Low, very soft fruit; high, very firm fruit	Low–high
	Bumpiness	Amount of ridges and bumps on the exterior of the fruit	Low, very smooth peel; high, very bumpy/textured skin	Low–high
Aromas and flavors	Overall aroma intensity	Overall orthonasal intensity of aroma while peeling the fruit		Low–high
	Overall flavor intensity	Overall retronasal intensity of aroma while peeling the fruit		Low–high
	Orange	Aroma (orthonasal) and flavor (retronasal) intensity of orange	0.5 g orange essential oil (sun essentials) + 20 g water	Low–high
	Mandarin	Aroma (orthonasal) and flavor (retronasal) intensity of mandarin	0.3 g tangerine essential oil (Healing Solutions) + 16 g water	Low–high
	Lemon/Lime	Aroma (orthonasal) and flavor (retronasal) intensity of a lemon and lime combination	2 g zest, 2 g juice, and 8 g pulp from a Eureka lemon and lime	Low–high
	Grapefruit	Aroma (orthonasal) and flavor (retronasal) intensity of grapefruit	g zest, 8 g juice, and 16 g pulp from a grapefruit	Low–high
	Fruity	Aroma (orthonasal) and flavor (retronasal) intensity of fruity notes, like apple and cherry	2 tbl fruit cocktail (Del Monte)	Low–high
	Tropical	Aroma (orthonasal) and flavor (retronasal) intensity of tropical-like notes of pineapple, mango, and banana	1/4 cup pineapple orange guava juice blend (Meadow Gold)	Low–high
	Floral	Aroma (orthonasal) and flavor (retronasal) intensity of flowers, like orange blossom and lavender	20 mL orange blossom water (Carlo Enterprises)	Low–high
	Pine	Aroma (orthonasal) and flavor (retronasal) intensity of pine-like notes	2 g crushed needles from a white fir tree (*Abies concolor*)	Low–high
	Grass	Aroma (orthonasal) and flavor (retronasal) intensity of grass-like notes	1 g cut grass (*Poa pratensis*)	Low–high
	Waxy	Aroma (orthonasal) and flavor (retronasal) intensity of wax-like notes	1 crayon (Crayola), chopped into 1/2″ pieces	Low–high
	Fermented	Aroma (orthonasal) and flavor (retronasal) intensity of fermented citrus	1 rotted Clementine mandarin	Low–high
	Woody	Aroma (orthonasal) and flavor (retronasal) intensity of wood-like notes	One moth ball (CedarFresh)	Low–high
	Sweet	Taste associate with small sugars	30 mL syrup from a jar of canned mandarins in light syrup (Del Monte)	Low–high
	Sour	Taste associated with acids	30 mL fresh squeezed lemon juice	Low–high
	Bitter	Taste associate with bitterants	30 mL fresh squeezed grapefruit juice	Low–high
Peeling	Peelability	Ease of removing the peel from the fruit	Low, very difficult to peel; high, very easy to peel	Low–high
	Peel elasticity	Pliability of peel after removed from fruit	Low, very crumbly; high, very elastic	Low–high
	Peel thickness	Thickness of peel after removed from fruit	Low, ~1/8″ thick; high, ~1/2″ thick	Low–high
	Separability of segments	Ease of separating the segments from each other without tearing the membrane	Low, very difficult to separate; high, segments separate very easily	Low–high
Albedo	Quantity of albedo	Amount of albedo on the exterior of the fruit after peeling		Low–high
Interior appearance	Interior color	Color of the interior flesh of the fruit	Low, pale yellow; high, dark orange	Low–high
	Size of navel	Size of navel as it protrudes to the inside of the fruit.	Low, no interior navel; high, interior navel encompasses 1/3 of fruit	Low–high
	Plumpness	How plump the segments appear after separation from each other	Low, very shriveled; high, very plump or full segments	Low–high
	Juiciness	Amount of juice released when chewing a segment of the fruit	Low, very dry; high, very juicy	Low–high
	Firmness of membrane	Force required to break the membrane encasing the vesicles	Low, very easy to bite through; high, very difficult to bite through	Low–high
	Fibrousness	Level of force required to chew through the juice vesicles	Low, vesicles not clearly defined; high, vesicles clearly defined and firm	Low–high
	Astringency	Mouthfeel associated with astringent foods	1 cm × 1 cm × 1 cm cube of unripe hachiya persimmon	Low–high

### Consumer testing

Adults and children (7–12 years old) from the local (Davis, Woodland and Sacramento) community were recruited for the tests. Potential participants were screened for appropriate age, lack of allergies, and consumption frequency of citrus. The age of the children was determined based on cognitive abilities required to use hedonic scales, intensity scales, and other sensory measures.^34^ Children were accompanied by a parent/guardian at all times but seated at their own booth. The adults participating in the study completed an online questionnaire aimed to collect information regarding their demographics, consumption habits, and psychographics.

The tasting sessions took place in the Silverado Vineyards Sensory Theater of the Robert Mondavi Institute for Wine and Food Science at the University of California, Davis. In total, 193 adults and 69 children participated in the tasting. Each consumer was given a double-sided test ballot for evaluation of each sample, plain crackers, water, and napkins. The fruit were served as two one-sixth wedges cut from the same fruit in soufflé cups placed on the tray in a fully balanced sequential monadic order. Their tray provided arrows, matched with the testing ballots, to ensure they tasted in the order designed. This study was approved for the use of human subjects by the Institutional Review Board of the University of California, Davis and all consumers consented to participating in the tasting session.

Adult consumers rated their degree of liking on the 9-point hedonic scale for appearance, overall liking, flavor, and texture as well as the adequacy of sweetness, sourness, firmness and juiciness on a 5-point Just-About-Right scale. Children consumers rated their overall liking and liking of appearance, taste and texture of the fruit on a smaller, 7-point hedonic scale as well as the adequacy of the sweetness, sourness, firmness, and juiciness of the fruit on a 3-point Just-About-Right scale. Check-All-That-Apply attributes were also presented to the consumers with a combination of hedonic and descriptive attributes. CATA terms are shown in Supplementary Tables 1 and 2. Ballots used were based on past consumer citrus evaluation.^18,33^ The mix of liking, JAR, and CATA questions provided the opportunity for mixed consumer analyses and comparisons with the descriptive panel. These combinations have shown effective results in the past.^19,35^

### ^1^H nuclear magnetic resonance of non-volatile components

The frozen juices were allowed to thaw at room temperature before being centrifuged. A 500 µL of juice supernatant was filtered using an Amicon Ultra-0.5 Centrifugal Filter Unit with a 3 kD cut-off (MilliporeSigma, Burlington, MA), which had been previously washed with deionized water. Two hundred and seven microliters of filtrate was mixed with 27 µL of an internal standard of 5 mM 3-(trimethylsilyl)-1-propanesulfonic acid-d6 (DSS-d6) in >98% D_2_O (Chenomx, Edmonton, AB, Canada). The pH of the samples was adjusted to 6.8 ± 0.1 and 180 µL of sample was transferred to a 3 mm NMR tube. ^1^H-NMR spectra were acquired on a Bruker Avance 600 NMR spectrometer at 298 K using the Bruker “noesypr1d” pulse program as previously described.^36^ The resulting NMR spectra were processed and profiled using Chenomx NMR Suite v8.3 as described.^36^ Quantitation of each compound was achieved as described in Weljie et al.,^37^ based on the concentration of the DSS-d6 internal standard.

### Chemicals

Authentic GC standards were purchased from Sigma-Aldrich (St. Louis, MO), Molekula Group, LLC (Santa Ana, CA), Acros Organics (Pittsburgh, PA), and TCI America (Portland, OR).

### HS-SPME-GC-MS-O analysis of volatile components

Volatile compounds in the orange juice samples were extracted using headspace solid-phase microextraction (HS-SPME). A 5 mL aliquot of freshly squeezed orange juice was transferred into a 40 mL vial before the addition of 1.80 g NaCl and 2 µL octyl acetate (0.05 µg/µL in methanol, internal standard) into each sample. The juice samples were gently agitated using a stir bar and placed in a 40 °C water bath (Baxter Scientific Products, Cincinnati, OH, USA) at 200 rmp for 30 min. After equilibration, a Stableflex fiber (2 cm, 50/30 µm, Divinylbenzene/Carboxen^TM^/polydimethylsiloxane, Supelco, Bellofonte, PA, USA) was exposed in the vial headspace at 40 °C for 30 min. Volatile compounds were identified using a PerkinElmer Clarus 680 gas chromatograph (PerkinElmer, Waltham, MA) equipped with a PerkinElmer Clarus SQ 8T mass spectrometer (PerkinElmer). The mass spectrometer was operated in the electron impact ionization mode with an ionizing energy of 70 eV. A constant pressure of helium, the carrier gas, was set at 30 psi as calculated using PerkinElmer Swafer utility software (PerkinElmer). Chromatographic separation was achieved by TR-FFAP capillary column (30 m × 0.32 mm × i.d., 0.25 µm df; Chrompack, Mühlheim, Germany). Injection port temperature was set at 280 °C, and the carrier gas, helium, was set to a flow rate of 1.5 mL/min. The original oven temperature was set at 40 °C for 2.0 min, then gradually increased to 230 °C at a rate of 5 °C /min, and finally held at 230 °C for 10 min. The scan range of the mass spectrometer was *m*/*z* 50 to 300. Aroma-active compounds were directly detected by the sniffing port of the GC-MS-O. Data collection was performed using TurboMass (v6.1.0, PerkinElmer). Peak identification of volatile compounds was achieved by comparing the linear retention index (LRI) values and mass spectra with the NIST library (National Institute of Standards and Technology, Gaithersburg, MA, USA). A mixture of n-alkane standards (C7-C30) was also analyzed to calculate retention indices. Additionally, authentic standards were run on a TR-FFAP column using their retention times to confirm compound identity. To evaluate the amount of each volatile in Navel oranges, a semi-quantification method was conducted using analyte/internal standard peak area ratio, based on the concentration of internal standard.

### Data analysis

The level of alpha was set at 0.05 for all statistical parameters. All data analysis was performed using R (Version 3.5.2, R Core Team, Vienna, Austria).

Chemical data for volatile compounds and non-volatile compounds were evaluated individually by analysis of variance (ANOVA). Any compound that did not differ significantly (at *P* ≤ 0.05) across the fruit was removed from further analysis. To compare with consumer cluster preferences, the compounds intensities were scaled, centered, and hierarchically clustered using Euclidean distances and Ward’s method.^38^ This effectively grouped the chemical compounds into clusters and reduced multi-collinearity in the dataset. The method of clustering chemical attributes is based on past analysis of red wine.^39^ Principal component analysis was also performed on the significant, scaled chemical values. More detailed information regarding the *F-*values and effect sizes of the chemical compounds is shown in Supplementary Table 4.

For the descriptive analysis data, a three-factor MANOVA (judges, replications, products) was performed on all attributes and followed with three-factor ANOVAs on each attribute using a pseudo-mixed model to test for product significance.^40^ The pseudo-mixed model uses judge by product and replication by product interactions as the denominator when testing for product effect significance. Fisher’s least significant difference (LSD) test followed ANOVA to separate the means for the different Navel oranges using the *agricolae* package (v1.2-8). More detailed statistical information regarding *F* values and effect size are shown in Supplementary Table 3.

For the consumer data, univariate statistics were performed on the hedonic questions. As is often done with consumer liking data measured on the 9-point hedonic scale, ANOVA and Fisher’s LSD were used to determine differences in overall liking^17^ and then principal component and cluster analyses were performed for preference mapping purposes. Just-About-Right data were compared across clusters or products using rating proportions and mean drop penalty analysis.^19^ CATA scores were analyzed across products using Cochran’s *Q*-test^41^ using the the RVAideMemoire package (v0.9-63-3). More detail regarding the *Q* values for the CATA attributes for the adult consumers is shown in Supplementary Table 5. For preference clustering, values for overall liking values were scaled and a Euclidean distance matrix was computed between the consumers. The consumers were then clustered according to Ward’s Method.^38^ The clusters were validated using a two-way ANOVA with cluster and product as main effects.

PLS regressions (PLS1 and PLS2) were employed for to model the dependence of consumer and descriptive analysis data onto chemical data and were generated using the plsdepot package (v0.1.17) with a ggplot2 (v3.1.0) wrapper.

### Reporting summary

Further information on research design is available in the Nature Research Reporting Summary linked to this article.

## Supplementary information

Supplementary Material.

reporting summary

## Data Availability

The data that support the findings of this study are available from the Citrus Research Board of California but restrictions apply to the availability of these data, which were used under license for the current study, and so are not publicly available. Data are however available from the authors upon reasonable request and with permission of Citrus Research Board of California.

## Supplementary information

**Supplementary information** accompanies the paper on the *npj Science of Food* website (10.1038/s41538-019-0055-7).
