# Supplementary material for: Chemical and sensory analysis of commercial Navel oranges in California
Source: NPJ Sci Food. 2019 Oct 30;3:22. doi: 10.1038/s41538-019-0055-7 (PMC6821911; doi:10.1038/s41538-019-0055-7)
Supplement: Supplementary file 1 — Supplementary Material. [file 41538_2019_55_MOESM1_ESM.docx]

Supplementary Table 1. Check-all-that-apply terms used by the adult consumers (n=193 adults) to evaluate the seven Navel orange samples. The terms were based on the descriptive analysis attributes generated by the trained judges.

| Adult Check-All-That-Apply terms | | |
| --- | --- | --- |
| Good Appearance | Flavorful | Balanced Flavor |
| Bad Appearance | Typical Orange Flavor | Bland |
| Blemished Peel | Other citrus flavor (lemon, lime, grapefruit) | Watery |
| Lots of pith (white albedo) | Tropical Flavor | Juicy |
| Aromatic | Fresh | Mushy |
| Sweet Tasting | Floral Flavor | Chewy |
| Sour Tasting | Grassy Flavor | Fibrous |
| Bitter Tasting | Complex Flavor |  |

Supplementary Table 2. Check-all-that-apply terms used by the child consumers (n=69 children) to evaluate the seven Navel orange samples. The terms were designed to be a simplified list of descriptive analysis attributes generated by the trained judges.

| Child Check-All-That-Apply terms | |
| --- | --- |
| Looks Good | Flavorful |
| Looks Bad | Watery |
| Sweet | Juicy |
| Sour | Dry |
| Bitter | Mushy |
| Yucky | Bland |
| Tasty | Chewy |

Supplementary Table 3. Descriptive analysis F values and effect sizes for all attributes - Only attributes deemed significant by a Psuedo-Mixed test were included for analysis - Each judge (n = 13) completed three replicates on the seven orange products. Attributes prepended with an “A” or an “F” are aroma and flavor attributes, respectively.

| Attribute | F value (product) | F value (Product x Replication) | F value (Product x Judge) | partial eta Squared (product) |
| --- | --- | --- | --- | --- |
| Size | 26.751 | 1.193 | 2.408 | 0.527 |
| Blemished | 2.177 | 0.534 | 5.193 | 0.083 |
| ExtColorhue | 12.487 | 1.841 | 3.129 | 0.342 |
| ColorUnif | 4.071 | 1.033 | 2.922 | 0.145 |
| ExtNavelSize | 5.427 | 1.071 | 8.367 | 0.184 |
| FruitFirmness | 8.224 | 1.116 | 3.184 | 0.255 |
| Bumpiness | 16.243 | 1.255 | 3.759 | 0.404 |
| AAromaInt | 5.151 | 1.447 | 0.855 | 0.177 |
| AOrange | 1.127 | 1.009 | 1.858 | 0.045 |
| AMandarin | 0.942 | 1.544 | 1.775 | 0.038 |
| ALemon/Lime | 4.482 | 1.665 | 0.466 | 0.157 |
| AGrapefuit | 1.797 | 1.360 | 1.207 | 0.070 |
| AFruity | 1.910 | 0.993 | 0.708 | 0.074 |
| ATropical | 2.204 | 1.505 | 0.662 | 0.084 |
| AFloral | 2.833 | 1.485 | 1.221 | 0.106 |
| AChemical | 4.187 | 1.004 | 0.695 | 0.149 |
| APine | 2.564 | 1.002 | 0.763 | 0.097 |
| AGrassy | 1.180 | 1.035 | 0.614 | 0.047 |
| AWoody | 2.807 | 1.291 | 1.066 | 0.105 |
| AWaxy | 1.148 | 1.423 | 0.511 | 0.046 |
| AWermented | 0.965 | 0.638 | 1.602 | 0.039 |
| Peelability | 6.454 | 0.630 | 0.444 | 0.212 |
| Peelelasticity | 2.209 | 1.179 | 0.654 | 0.084 |
| PeelThickness | 6.365 | 1.337 | 3.546 | 0.210 |
| SegmentSep | 3.596 | 0.798 | 1.063 | 0.130 |
| AlbedoQuantity | 5.857 | 1.393 | 1.233 | 0.196 |
| IntColor | 3.813 | 1.144 | 1.356 | 0.137 |
| IntNavelSize | 7.508 | 1.012 | 2.588 | 0.238 |
| Plumpness | 0.619 | 1.122 | 1.008 | 0.025 |
| Sweet | 8.702 | 1.325 | 0.672 | 0.266 |
| Sour | 5.819 | 1.617 | 1.367 | 0.195 |
| Bitter | 3.318 | 1.799 | 1.568 | 0.121 |
| FOverall | 7.738 | 1.227 | 0.680 | 0.244 |
| FOrange | 4.337 | 1.286 | 0.447 | 0.153 |
| FMandarin | 2.104 | 2.030 | 1.398 | 0.081 |
| FLemon/lime | 2.097 | 0.965 | 0.628 | 0.080 |
| FGrapefruit | 0.684 | 1.153 | 0.625 | 0.028 |
| FFruity | 4.694 | 1.064 | 1.302 | 0.164 |
| FTropical | 0.856 | 0.761 | 1.231 | 0.034 |
| FFloral | 1.899 | 1.466 | 0.620 | 0.073 |
| FPine | 1.133 | 0.958 | 0.842 | 0.045 |
| FGrassy | 0.779 | 0.798 | 0.502 | 0.031 |
| FWoody | 1.406 | 0.613 | 0.607 | 0.055 |
| FWaxy | 0.938 | 0.892 | 1.066 | 0.038 |
| FFermented | 1.827 | 0.954 | 0.694 | 0.071 |
| Juiciness | 11.860 | 1.137 | 0.790 | 0.331 |
| MembraneFirm | 3.928 | 1.288 | 2.345 | 0.141 |
| Fibrousness | 9.154 | 1.264 | 0.992 | 0.276 |
| Astringent | 1.636 | 0.722 | 0.692 | 0.064 |

Supplementary Table 4. ANOVA F values and partial eta squared values for the chemical compounds - Volatile compounds measure by gas chromatography (GC) were measured in triplicate while measurements for the Nuclear Magnetic Resonance (NMR) were measured 10 times - Only compounds with a significant (P≤0.05) F value were selected for further analysis

| Compound | F value (product) | | partial eta squared (product) | | GC or NMR | | |
| --- | --- | --- | --- | --- | --- | --- | --- |
| Methyl.butanoate | | 1.932 | | 0.453 | | GC |  |
| α.Pinene | | 1.246 | | 0.348 | | GC |  |
| Ethyl.butanoate | | 0.675 | | 0.224 | | GC |  |
| Hexanal | | 2.915 | | 0.555 | | GC |  |
| (-)-β-Pinene | | 1.717 | | 0.424 | | GC |  |
| β.Myrcene | | 1.440 | | 0.382 | | GC |  |
| D.Limonene | | 1.635 | | 0.412 | | GC |  |
| Ethyl.Hexanoate | | 2.323 | | 0.499 | | GC |  |
| Octanal | | 2.159 | | 0.481 | | GC |  |
| 6-Methyl-5-heptene-2-one | | 1.942 | | 0.454 | | GC |  |
| Nonanal | | 0.455 | | 0.163 | | GC |  |
| (E )-2-Octen-1-al | | 0.917 | | 0.282 | | GC |  |
| p.Cymenene | | 0.244 | | 0.095 | | GC |  |
| Linalool | | 4.577 | | 0.662 | | GC |  |
| (-)-Terpinen-4-ol | | 1.146 | | 0.329 | | GC |  |
| 2-Oxoglutarate | | 3.519 | | 0.251 | | NMR |  |
| 4-Aminobutyrate | | 14.981 | | 0.588 | | NMR |  |
| Adenosine | | 11.866 | | 0.531 | | NMR |  |
| Alanine | | 9.867 | | 0.484 | | NMR |  |
| Arginine | | 4.292 | | 0.290 | | NMR |  |
| Ascorbate | | 3.308 | | 0.240 | | NMR |  |
| Asparagine | | 1.115 | | 0.096 | | NMR |  |
| Aspartate | | 4.367 | | 0.294 | | NMR |  |
| Betaine | | 5.464 | | 0.342 | | NMR |  |
| Choline | | 23.356 | | 0.690 | | NMR |  |
| Citrate | | 3.879 | | 0.270 | | NMR |  |
| Cytidine | | 5.157 | | 0.329 | | NMR |  |
| Ethanol | | 12.129 | | 0.536 | | NMR |  |
| Formate | | 8.978 | | 0.461 | | NMR |  |
| Fructose | | 6.405 | | 0.379 | | NMR |  |
| Galactose | | 5.639 | | 0.349 | | NMR |  |
| Glucose | | 7.265 | | 0.409 | | NMR |  |
| Histidine | | 0.766 | | 0.068 | | NMR |  |
| Isoleucine | | 12.018 | | 0.534 | | NMR |  |
| Leucine | | 15.773 | | 0.600 | | NMR |  |
| Limonin glucoside | | 4.690 | | 0.309 | | NMR |  |
| Lysine | | 3.858 | | 0.269 | | NMR |  |
| Malate | | 6.159 | | 0.370 | | NMR |  |
| Methanol | | 11.879 | | 0.531 | | NMR |  |
| Phenylalanine | | 4.798 | | 0.314 | | NMR |  |
| Proline | | 13.389 | | 0.560 | | NMR |  |
| Succinate | | 11.659 | | 0.526 | | NMR |  |
| Sucrose | | 3.927 | | 0.272 | | NMR |  |
| Threonine | | 5.466 | | 0.342 | | NMR |  |
| Trigonelline | | 2.311 | | 0.180 | | NMR |  |
| Tyrosine | | 9.002 | | 0.462 | | NMR |  |
| Uridine | | 5.854 | | 0.358 | | NMR |  |
| Valine | | 13.683 | | 0.566 | | NMR |  |
| Myo-Inositol | | 5.432 | | 0.341 | | NMR |  |
| Proline betaine | | 8.465 | | 0.446 | | NMR |  |

Supplementary Table 5. Q statistic values for the Check-All-That-Apply attributes evaluated by the adult consumers (n= 193 adults, age 18+) using Cochran’s Q test for the seven Navel orange samples.

| CATA Attribute | Q statistic |
| --- | --- |
| Good_Appearance | 36.213 |
| Bad_Appearance | 10.979 |
| Blemished_Peel | 38.613 |
| Lots_of_pith_(white_albedo) | 12.913 |
| Aromatic | 26.267 |
| Sweet_Tasting | 43.341 |
| Sour_Tasting | 47.214 |
| Bitter_Tasting | 16.054 |
| Flavorful | 74.538 |
| Typical_Orange_Flavor | 23.869 |
| Other_citrus_flavor_(lemon_lime_grapefruit) | 7.983 |
| Tropical_Flavor | 20.788 |
| Fresh | 34.465 |
| Floral_Flavor | 6.909 |
| Grassy_Flavor | 2.868 |
| Complex_Flavor | 7.857 |
| Balanced_Flavor | 30.542 |
| Bland | 76.807 |
| Watery | 44.610 |
| Juicy | 56.900 |
| Mushy | 22.863 |
| Chewy | 16.155 |
| Fibrous | 25.506 |

Supplementary Table 6. Child liking correlation between sensory modalities^1^. Children (n=69, age 7-12) evaluated the seven orange samples for liking of the four different attributes on the 7-point hedonic scale.

| Attribute | **Overall Liking** | **Appearance** | **Taste** | **Texture** |
| --- | --- | --- | --- | --- |
| **Overall Liking** | - | **0.38** | **0.83** | **0.56** |
| **Appearance** | **0.38** | - | **0.29** | **0.35** |
| **Taste** | **0.83** | **0.29** | - | **0.49** |
| **Texture** | **0.56** | **0.35** | **0.49** | - |

^1^All values are significant (P≤0.05) by Pearson’s correlation test

Supplementary Table 7. Adult liking correlation between sensory modalities^I^. The adults (n=193, age 18+) evaluated the seven Navel orange samples for the five attributes using the 9-point hedonic scale.

| Attribute | **Overall Liking** | **Appearance** | **Internal Color** | **Texture** | **Flavor** |
| --- | --- | --- | --- | --- | --- |
| **Overall Liking** | - | **0.36** | **0.38** | **0.74** | **0.90** |
| **Appearance** | **0.36** | - | **0.68** | **0.35** | **0.30** |
| **Internal Color** | **0.38** | **0.68** | - | **0.40** | **0.34** |
| **Texture** | **0.74** | **0.35** | **0.40** | - | **0.67** |
| **Flavor** | **0.90** | **0.30** | **0.34** | **0.67** | - |

^1^All values are significant (P≤0.05) by Pearson’s correlation test.

Supplementary Table 8. Check-All-That-Apply rating proportions for the seven different Navel oranges as rated by the adult consumers (n=193, age 18+)^1^

| **Attribute** | Navel A | Navel B | Navel F | Navel OL | Navel S | Navel SW | Navel W |  |
| --- | --- | --- | --- | --- | --- | --- | --- | --- |
| **Good Appearance** | 0.61 | 0.73 | 0.73 | 0.72 | 0.65 | 0.65 | 0.52 |  |
| **Bad Appearance** | 0.11 | 0.05 | 0.06 | 0.08 | 0.08 | 0.08 | 0.11 |  |
| **Blemished Peel** | 0.19 | 0.03 | 0.06 | 0.10 | 0.12 | 0.08 | 0.11 |  |
| **Lots of pith (white albedo)** | 0.24 | 0.13 | 0.17 | 0.18 | 0.20 | 0.15 | 0.22 |  |
| **Aromatic** | 0.32 | 0.22 | 0.29 | 0.22 | 0.27 | 0.19 | 0.18 |  |
| **Sweet Tasting** | 0.60 | 0.46 | 0.53 | 0.45 | 0.41 | 0.41 | 0.32 |  |
| **Sour Tasting** | 0.15 | 0.04 | 0.15 | 0.26 | 0.21 | 0.17 | 0.13 |  |
| **Bitter Tasting** | 0.03 | 0.04 | 0.07 | 0.05 | 0.10 | 0.07 | 0.10 |  |
| **Flavorful** | 0.48 | 0.29 | 0.49 | 0.35 | 0.33 | 0.35 | 0.16 |  |
| **Typical Orange Flavor** | 0.52 | 0.38 | 0.48 | 0.47 | 0.47 | 0.52 | 0.37 |  |
| **Other citrus flavor (lemon, lime, grapefruit)** | 0.07 | 0.05 | 0.04 | 0.10 | 0.07 | 0.06 | 0.09 |  |
| **Tropical Flavor** | 0.13 | 0.12 | 0.18 | 0.11 | 0.08 | 0.08 | 0.06 |  |
| **Fresh** | 0.51 | 0.38 | 0.51 | 0.42 | 0.42 | 0.44 | 0.31 |  |
| **Floral Flavor** | 0.07 | 0.07 | 0.11 | 0.10 | 0.07 | 0.07 | 0.06 |  |
| **Grassy Flavor** | 0.04 | 0.04 | 0.05 | 0.03 | 0.06 | 0.04 | 0.06 |  |
| **Complex Flavor** | 0.11 | 0.07 | 0.12 | 0.10 | 0.11 | 0.06 | 0.09 |  |
| **Balanced Flavor** | 0.27 | 0.12 | 0.26 | 0.18 | 0.21 | 0.24 | 0.12 |  |
| **Bland** | 0.14 | 0.39 | 0.13 | 0.22 | 0.17 | 0.25 | 0.37 |  |
| **Watery** | 0.15 | 0.22 | 0.08 | 0.15 | 0.10 | 0.12 | 0.26 |  |
| **Juicy** | 0.70 | 0.51 | 0.65 | 0.58 | 0.56 | 0.54 | 0.40 |  |
| **Mushy** | 0.11 | 0.17 | 0.12 | 0.18 | 0.12 | 0.09 | 0.22 |  |
| **Chewy** | 0.10 | 0.10 | 0.12 | 0.16 | 0.15 | 0.08 | 0.17 |  |
| **Fibrous** | 0.15 | 0.20 | 0.11 | 0.15 | 0.24 | 0.16 | 0.24 |  |
| **^1^All attributes are significant different (p≤0.05) by Cochran’s Q-test** | | | | | | | | |
